# American College of Radiology Appropriateness Criteria®: a bibliometric analysis of panel members

**DOI:** 10.1186/s13244-023-01456-z

**Published:** 2023-07-03

**Authors:** Ajay Malhotra, Suryansh Bajaj, Tushar Garg, Mihir Khunte, Bhavya Pahwa, Xiao Wu, Seyedmehdi Payabvash, Suresh Mukherjee, Dheeraj Gandhi, Howard P. Forman

**Affiliations:** 1 Department of Radiology and Biomedical Imaging, Yale School of Medicine, Tompkins East 2, 333 Cedar St, Box 208042, New Haven, CT 06520-8042 USA; 2 Division of Vascular and Interventional Radiology, Russell H. Morgan Department of Radiology and Radiological Science, Johns Hopkins University School of Medicine, Baltimore, USA; 3 University College of Medical Sciences, Delhi, India; 4 Department of Radiology, University of California at San Francisco, San Francisco, USA; 5 Radiology and Radiation Oncology, University of Illinois, Peoria, IL and Robert Wood Johnson Medical School, Newark, NJ, USA; 6 Interventional Neuroradiology, Nuclear Medicine, Neurology and Neurosurgery, University of Maryland School of Medicine, Maryland, USA

**Keywords:** American College of Radiology, Appropriateness criteria, Appropriate use criteria

## Abstract

**Objective:**

To assess the features of panel members involved in the writing of the ACR-AC and identify alignment with research output and topic-specific research publications.

**Methods:**

A cross-sectional analysis was performed on the research output of panel members of 34 ACR-AC documents published in 2021. For each author, we searched Medline to record total number of papers (P), total number of ACR-AC papers (C) and total number of previously published papers that are relevant to the ACR-AC topic (R).

**Results:**

Three hundred eighty-three different panel members constituted 602 panel positions for creating 34 ACR-AC in 2021 with a median panel size of 17 members. Sixty-eight (17.5%) of experts had been part of ≥10 previously published ACR-AC papers and 154 (40%) were members in ≥ 5 published ACR-AC papers. The median number of previously published papers relevant to the ACR-AC topic was 1 (IQR: 0–5). 44% of the panel members had no previously published paper relevant to the ACR-AC topic. The proportion of ACR-AC papers (C/P) was higher for authors with ≥ 5 ACR-AC papers (0.21) than authors with < 5 ACR-AC papers (0.11, *p* < 0.0001); however, proportion of relevant papers per topic (R/P) was higher for authors with < 5 ACR-AC papers (0.10) than authors with ≥ 5 ACR-AC papers (0.07).

**Conclusion:**

The composition of the ACR Appropriateness Criteria panels reflects many members with little or no previously published literature on the topic of consideration. Similar pool of experts exists on multiple expert panels formulating imaging appropriateness guidelines.

**Key Points:**

- There were 68 (17.5%) panel experts on ≥ 10 ACR-AC panels.
- Nearly 45% of the panel experts had zero median number of relevant papers.
- Fifteen panels (44%) had > 50% of members having zero relevant papers.

## Introduction

The use of imaging studies in medicine has dramatically increased over the past two decades [1]. As patients receive medical care in multiple settings and hospitals, there is increased concern over ordering of imaging studies for indications that may not meet evidence-based guidelines [2]. Previous studies have shown that 20–50% of all the advanced imaging studies performed do not make a significant difference in patient care [3]. Clinical practice guidelines and education have been shown to be key factors in reducing low-value imaging [4]. In 2016, the Centers for Medicare & Medicaid Services (CMS) recognized ACR as a “qualified Provider-Led Entity” (qPLE), approving the American College of Radiology Appropriateness Criteria® (ACR-AC) as the Medicare Appropriate Use Criteria (AUC) [5]. Through this provision, providers are required to make a documented AUC consult using a CMS qualified clinical decision support system (CDSS) prior to ordering advanced imaging investigations like CT, MRI, PET scan, etc., to fulfill the Protecting Access to Medicare Act (PAMA) requirements for reimbursement of these studies by Medicare [6]. The ACR-AC are currently the most comprehensive national guidelines that aim to guide providers to use imaging modalities in the most appropriate and judicious manner and avoid inappropriate utilization of radiological imaging [7]. The European Society of Radiology (ESR) also adapted the ACR criteria for use in the European clinical decision support (CDS) platform ESR iGuide [7, 8].

The ACR-AC are developed and reviewed by “expert panels with leaders in radiology and other specialties” [7]. The methodology relies on combination of evidence, and when the data from scientific and technology assessment studies are insufficient, “expert consensus” [7]. As per the ACR, the methodology for evidence is based on the Grading of Recommendations Assessment, Development and Evaluation (GRADE) approach along with the National Institute of Medicine’s publication “Clinical Practice Guidelines We Can Trust” [7, 9]. The GRADE approach emphasizes grading the quality (or certainty) of evidence and strength of recommendations [10, 11]. GRADE recommends assessing the certainty in evidence for each important outcome using categories, and evidence summaries should be the basis for judgements about certainty in evidence and strength of recommendations [10, 11]. The ACR-AC provides recommendations in terms of appropriateness of imaging but does not provide the certainty in evidence for the readers. Lack of transparency about strength of evidence raises concern for dependence on the degree of “expert” opinion and consensus on the formulation of criteria.

Given the importance of ACR-AC in regulatory compliance and clinical decision-making, the aim of this study was to assess the scholarly expertise of panel members involved in the formation of the ACR-AC by looking at their past research productivity and topic-specific publications. We focused on the ACR-AC published in 2021, during which a total of 18 new and 13 revised topics were added to the ACR-AC.

## Materials and methods

We conducted a cross-sectional analysis of all the ACR-AC published in 2021. For each ACR-AC document, we extracted the name of the paper, author name(s), and their institutional affiliation(s). Once the information from the ACR-AC document was extracted, we searched the PubMed database to record the total number of papers (P), the total number of ACR-AC papers (C), the total number of first and non-first author papers relevant to the ACR-AC topic (R) for each listed author in the ACR-AC document. To search the previously published papers relevant to the ACR-AC topic, keywords were systematically selected by including the keywords from the ACR-AC document as well as from all the clinical variants described in the respective AC paper. The search was conducted in March 2022 by two independent authors (S.B., T.G.) with 20% overlap (reviewed independently by both) and any conflicting information in the data was resolved after discussion with the senior author (A.M.). In May 2022, a third author (M.K.) independently extracted the data for 7 out of 34 panels for cross-verification. The data were collected in Microsoft Excel (Redmond, WA) and analyzed using STATA/ BE Version 17 (StataCorp, College Station, TX).

### Statistical analysis

Summary statistics were used to analyze the results of this study. Categorical measures were summarized using rates and/or counts. Number of papers was analyzed using median and interquartile range because the data were not normally distributed as determined by the Shapiro–Wilk Normality test. The proportions were calculated by dividing the total number of ACR-AC papers and relevant papers to the total number of papers found on PubMed database for each author. Logistic regression was used to calculate the normal 95% confidence intervals for each demographic group. Differences between the two groups (≥ 5 ACR-AC papers, < 5 ACR AC papers) were evaluated using the Student’s t test. Univariate followed by multivariate linear regression models were used to identify and confirm correlations and predictive factors for the total number of ACR-AC papers by each unique author.

## Results

**Fig. 1:**
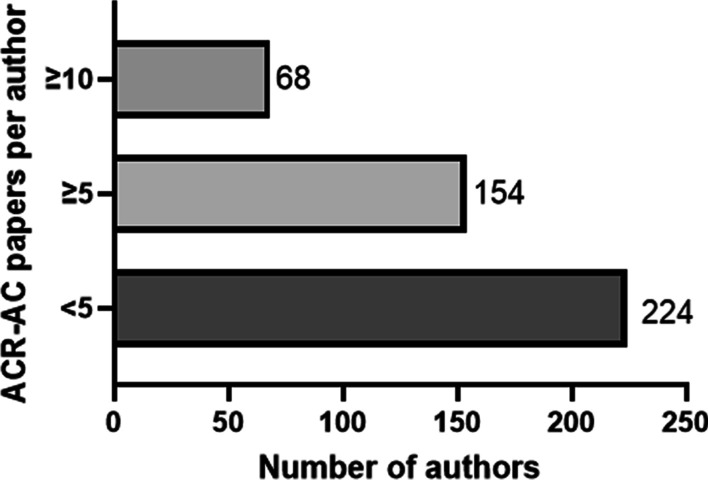
Distribution of authors based on number of previously published ACR-AC papers

A total of 34 ACR-AC published in 2021 were included in the study with a total panel size of 602 members that included 383 discrete panel members. Of the 602 panel positions, 468 (77.7%) positions were filled by radiology and 134 (22.3%) by experts from other specialties. The panel size ranged from 12 to 22 members with a median size of 17 members per panel. 68 out of 383 (17.5%) panel members had ≥ 10 previously published ACR-AC papers and 154 (40%) had ≥ 5 published ACR-AC papers (Fig. 1). The median total number of PubMed indexed publications for each author in the cohort was found to be 45 (IQR: 24–97, range: 3–359) and median total number of ACR-AC papers was 4 (IQR: 2–9, range: 1–27). The median number of previously published papers relevant to the ACR-AC topic was 1 (IQR: 0–5, range: 0–96). The relevant papers (R) were further stratified into first author papers (median: 0, IQR: 0–5, range: 0–96) and non-first author papers (median: 1, IQR: 0–4, range: 0–88). The panel members were the first authors on 25% of the relevant papers. 44.7% (269/602) of the panel members had no previously published paper relevant to the ACR-AC topic.

**Fig. 2:**
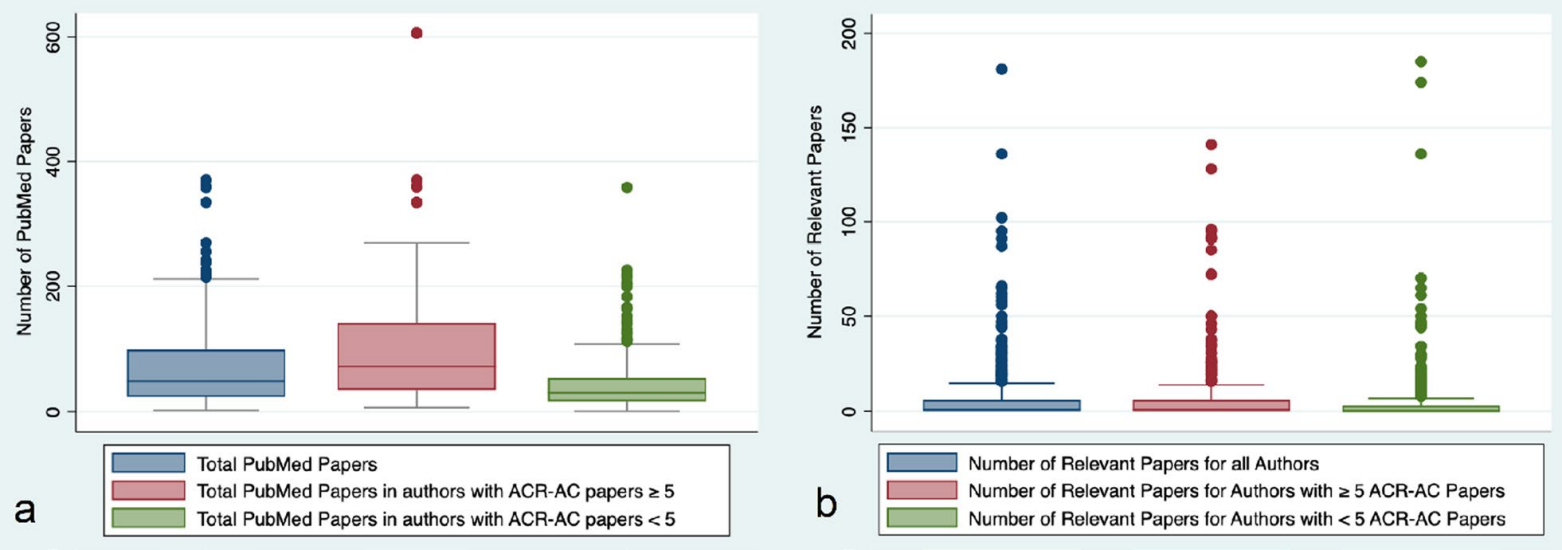
Box plot graphs demonstrating comparative analysis of the entire cohort of authors with subgroups having ≥ 5 ACR-AC papers and < 5 ACR-AC papers in terms of: **a** Total number of PubMed papers, and (**b**) Total number of relevant papers per ACR-AC topic per author

**Fig. 3:**
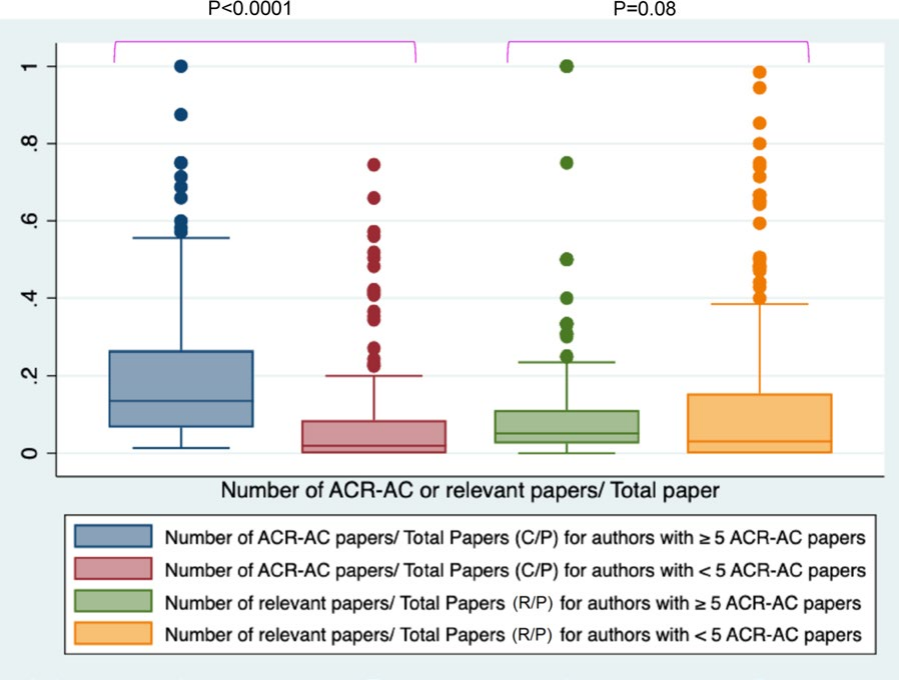
Box plot graph demonstrating comparison of C/P and R/P ratios of authors having ≥ 5 ACR-AC papers with those having < 5 ACR-AC papers

When compared to authors with < 5 ACR AC papers (229/374, 60%), authors with ≥ 5 ACR-AC papers (154/374, 40%) had a higher number of total PubMed publications (68, IQR: 36–138, vs. 31 IQR: 17–61.5), and higher number of relevant papers per assigned panel topic (1, IQR: 0–6 vs. 0, IQR: 1–2) (Fig. 2). The ratio of total ACR-AC papers by each author to the total number of papers by the author (C/P) was 0.16 (95% CI 0.15–0.18) and the proportion of relevant papers by the author to the total number of papers (R/P) was 0.08 (95% CI 0.07–0.10). Ten authors had ACR AC paper constitute ≥ 75% of all their publications (C/P ratio), 24 authors had ≥ 50%, 35 had ≥ 40%, 50 had ≥ 30%, and for 86 authors this was ≥ 20%. The proportion of the ACR-AC papers (C/P) was higher for authors with ≥ 5 ACR-AC papers (0.21, CI 0.18–0.24) than for authors with < 5 ACR-AC papers (0.11, CI 0.08–0.13, *p* < 0.0001); however, the proportion of relevant papers per topic (R/P) was higher for authors with < 5 ACR-AC papers (0.10, CI 0.08–0.12) than authors with ≥ 5 ACR-AC papers (0.07, CI 0.06–0.09), although this difference was not statistically significant (*p* = 0.08) (Fig. 3). While the univariate analysis showed a significant association of the total number of previously published papers (*p* = 0.0001) and the number of relevant papers (*p* = 0.011) with the total number of ACR-AC papers, on multivariate linear regression, only the total number of previously published papers had a significant and independent association (*p* = 0.0001).

**Fig. 4:**
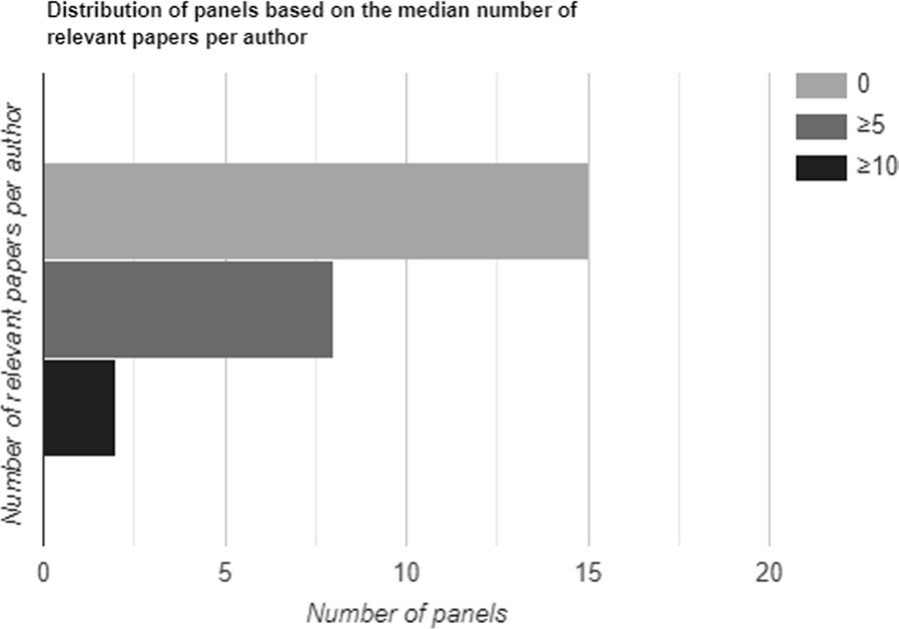
Distribution of panels based on the median number of relevant papers per author

**Fig. 5:**
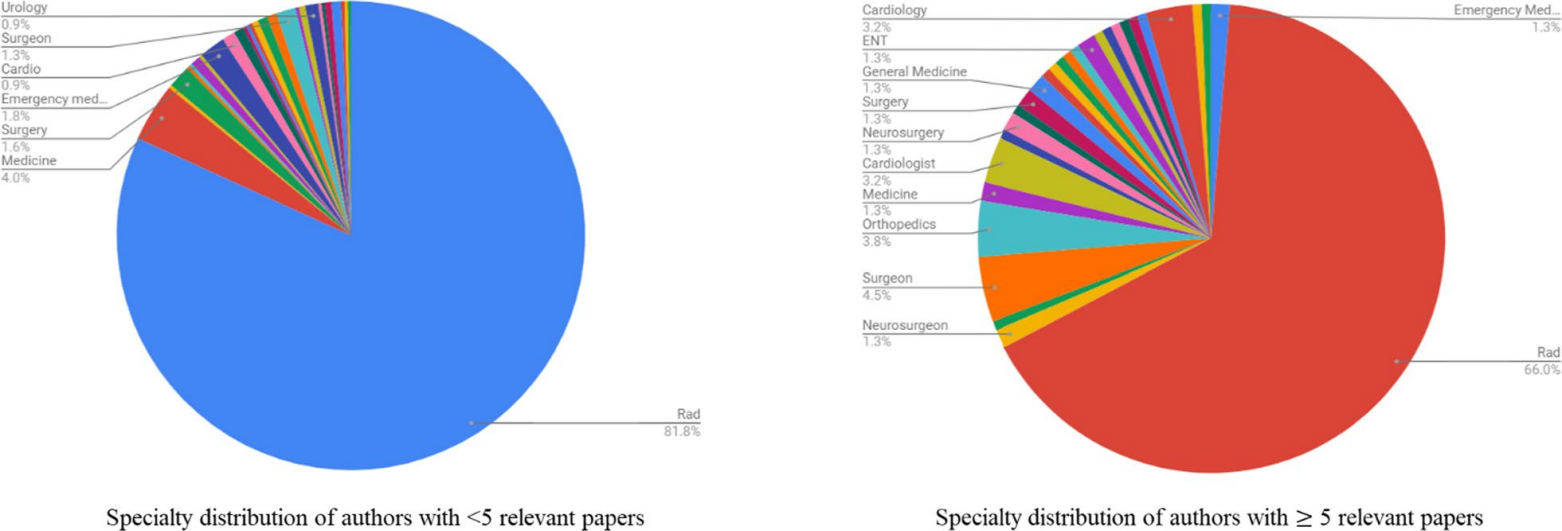
Distribution of authors based on specialty and number of relevant papers

Fifteen panels out of 34 (44%) had a median number of zero relevant papers per author and eight panels (23.5%) had a median of ≥ 5 relevant papers per author. Two panels (6%) had ≥ 10 median number of relevant papers per author (Fig. 4). Both the panels having ≥ 10 median number of relevant papers were in breast radiology—Breast Cancer Screening and Imaging of the Axilla. Fifteen panels (44%) had > 50% of members having zero relevant papers. Ten panels (29.4%) had < 10% of members having ≥ 5 relevant papers and five (14.7%) had zero members having ≥ 5 relevant papers. Only nine panels (26.5%) had > 50% of members having ≥ 5 relevant papers. A higher proportion of non-radiology experts were found to have a greater number of relevant papers with 40% (53/134) having ≥ 5 relevant papers per author per panel topic when compared to the radiology experts (22%, 103/468). Radiology experts constituted 81.8% of the authors having < 5 relevant papers (365/446) while for the authors with ≥ 5 relevant papers, radiology experts constituted 66% (103/156) of the positions (Fig. 5). Of the 103 radiology experts having ≥ 5 relevant papers, 28% (29/103) were authors on the three panels of breast radiology.

## Discussion

The ACR adopted the characteristics for developing acceptable medical practice guidelines developed by the Institute of Medicine (now, National Academy of Medicine) and used by the Agency for Healthcare Research and Quality (AHRQ) which emphasizes the maximal possible use of scientific evidence, while also recognizing that the expert panel consensus is needed in scenarios where sufficient literature is not available [12]. The methodology for creating the ACR-AC is based on the RAND/UCLA guidelines that involve rating the appropriateness of the use of imaging in predefined clinical variants using modified Delphi rounds to reach a consensus recommendation [12]. RAND-UCLA manual recommends that the main selection criteria to be considered are acknowledged leadership in the panel’s member specialty. The importance of having diverse panels with members having specific expertise in different aspects of the assigned topic is emphasized [7, 13]. While the ACR methodology subcommittee has described the process of developing ACR-AC in detail, no previous study has evaluated the expert panel structure and composition to our knowledge. This study assessed the panel members involved in the development of the ACR-AC by performing a bibliometric analysis of their prior literature contributions.

Our study included 34 ACR-AC papers that were released in 2021. Six hundred two panel positions comprised 383 experts with a median panel strength of 17. The panel members had a median of 45 prior publications, however, the median number of relevant papers previously published by the panel members was only 1, with nearly 44% of the authors not having any prior publications on the topic of their assigned panel. Nearly half (16/34, 47%) of the panels had zero median number of relevant papers. 44% (15/34) of the panels had > 50% of members having zero relevant papers. Additionally, 17.5% of the experts were members of more than ten panels and 40% were members of more than five panels with the panel members being on a median number of four panels. These results show that a similar pool of experts is serving on multiple panels which may be a potential source of bias in the development of the AC. For experts serving on multiple panels, their work on the respective topics assigned to different panels was less compared to the experts who were on less than five panels. Additionally, the ACR-AC documents constitute 16% of the experts’ total publications while the relevant publications accounted for only 8% of their prior research output. These results are further potentially compromised with the experts on more than five panels having a higher proportion of ACR-AC publications (21% vs. 11%, *p* < 0.001) but a lower proportion of relevant papers (7% vs. 10%, *p* = 0.08) than their counterparts on less than five panels.

Concerns about influential professional society guidelines written by insiders have been previously expressed [14]. Joining these guidance panels is a means to advance visibility and career within the specific medical specialty [14]. Practice guidelines tend to be frequently cited, and practice guidelines are particularly helpful in promoting careers of specialists as well as impact factors of specialty journals [14]. Concerns about the utility of practice guidelines to improve medicine, versus homogenizing biased, collective and organized ignorance have been recently raised [14].

While we believe that the years of experience and leadership qualities of some of the experts can potentially add value to multiple panels, it is important to recognize that the ACR-AC are created using a consensus method based on the experts’ clinical expertise. The presence of the same providers on different panels can limit the scope of inclusivity of different opinions and judgments, thereby leading to potential bias. It can also undermine the rigorous scrutiny of the recruitment process of the panel members.

Well-balanced multidisciplinary expert panels from a diversity of practice settings are supposed to be critical selection criteria for the RAND-UCLA Delphi method used by the ACR for defining ACR-AC [13]. However, we found that nearly all of the panel members in the 2021 ACR-AC are radiologists working in academic, teaching hospitals (detailed data available on request). 22.3% of panel positions were filled by experts from other specialties and had significantly higher relevant papers to the topic relative to radiologists overall. Multidisciplinary collaborations with other societies and the inclusion of established experts on relevant topics lends to greater credibility and likely acceptance of ACR-AC.

In addition to selecting radiology panel members based on their society affiliations or administrative positions, it may be prudent for ACR to consider prior contributions to the specific topic assigned to the panels. The expertise of the panel members may be especially relevant if the quality of the evidence is low on a specific topic. It has previously been shown that when there is little evidence from well-conducted clinical trials, the reliability of the panel process is likely to be low [13].

Although the ACR-AC Methodology describes the strength of evidence assessment in their manual, this information is not publicly available to the readers as recommended by GRADE [10, 11]. The literature reviews for appropriateness studies are typically less strict in their inclusion criteria compared to Cochrane reviews [13]. Although RAND-UCLA criteria recommend that the search strategy used should be documented in the final report to help the reader judge if the literature review is based on adequate body of evidence, this information is generally not included in the ACR-AC. The questionnaire used for the Delphi method iterative process is also recommended to be made public but is currently not available. We find that a significant proportion of studies used as evidence are from cohort studies or case series. High-quality systematic reviews, if available, are recognized to constitute a substantial contribution to the available evidence. Although a detailed analysis of the included ACR-AC for this was outside the scope of the current study, the authors recognize that at least some ACR-AC did not include these even when available in the last 5 years [15–17].

Comparison to other society guidelines would be helpful in understanding their process, and ways to improve radiology guidelines. The American Heart Association/ American Stroke Association (AHA/ASA) also issues guidelines relevant to the specialty [18]. These guidelines present the Level (Quality) of Evidence from Level A (based on randomized trials) to Level C (Expert Opinion). AHA/ASA guidelines also routinely present the Class (Strength) of Recommendation as Class I (Strong), Class IIa (Moderate), Class IIb (Weak), Class III (No Benefit or Harm). Comparative Effectiveness studies constitute an important basis for these recommendations. In contrast, much of radiology guidelines focus on imaging efficacy (imaging acquisition and diagnostic accuracy) and not on effectiveness (patient outcomes and quality of life). Although beyond the scope of this study, we found that ACR-AC often have not even considered or mentioned studies focusing on eventual health outcomes or ultimate societal benefit [15, 16, 19, 20]. There is an increased focus on establishing the role of radiology in value-based health care [21]. Including a discussion on effectiveness relevant to the topic would provide readers a greater sense of evidence in current literature on effectiveness forming the basis for the recommendations, as well as highlight the areas that need more research. Correlating the imaging guidelines with the clinical practice guidelines would also be critical to remove inconsistencies and needs further study.

A review of 139 clinical practice guidelines in 2018 also found low percentages of guidelines reporting important aspects of authorship, panel selection process/criteria and characteristics of individual authors [22]. External review and quality assurance, as recommended by RIGHT (Reporting Items for Practice Guidelines in Healthcare) Working Group are also lacking [23].

The methodology for the ACR Appropriateness criteria is the basis for the work of ESR Referral Guideline Subcommittee (RGSC) and is adhered to as far as possible. However, additional guidance principles have been established emphasizing that any changes or additional guidelines should be evidence-based to the extent possible and expert opinion should only function as a supplement when necessary.

Our study has several limitations. While prior research output is one way to evaluate a member’s expertise, it is also important to consider that members with years of clinical experience without significant research output can add value and diversify the scope of the panels. Secondly, as per ACR-AC methodology, the expert panels may include research authors, who may be members in training including radiology residents and fellows as well as attending physicians. The stated goal is to provide them with mentoring and early exposure to the process of creating evidence-based guidelines. However, it is unclear how this is consistent with the RAND-UCLA Appropriateness method or with the stated objective of having “expert” panel members. Lastly, we did not classify the previous relevant papers into Scientific papers, review articles, case reports or commentary/opinions and included all types of PubMed publications based on the keywords.

In conclusion, the ACR-AC are intended to be a helpful aid for physicians to optimize the use of imaging in different clinical scenarios. The importance of ACR-AC cannot be overstated given their national implementation under the medical appropriateness use criteria program. Our study provides the basis to reconsider staffing decisions when forming expert panels that will ultimately influence the healthcare delivery for millions of Americans every year.

## Data Availability

All relevant data can be made available on reasonable request to the Corresponding author.

## Abbreviations

ACR AC: American College of Radiology Appropriateness Criteria®
AUC: Appropriate use criteria
GRADE: Grading of recommendations assessment, development and evaluation
